# Hepatitis C virus NS3/4A inhibitors and other drug-like compounds as covalent binders of SARS-CoV-2 main protease

**DOI:** 10.1038/s41598-022-15930-z

**Published:** 2022-07-16

**Authors:** Babak Andi, Desigan Kumaran, Dale F. Kreitler, Alexei S. Soares, Jantana Keereetaweep, Jean Jakoncic, Edwin O. Lazo, Wuxian Shi, Martin R. Fuchs, Robert M. Sweet, John Shanklin, Paul D. Adams, Jurgen G. Schmidt, Martha S. Head, Sean McSweeney

**Affiliations:** 1 Center for BioMolecular Structure, NSLS-II, Brookhaven National Laboratory, Upton, NY 11973 USA; 2 Biology Department, Brookhaven National Laboratory, Upton, NY 11973 USA; 3 Molecular Biophysics and Integrated Bioimaging Division, Lawrence Berkeley National Laboratory, Berkeley, CA 94720 USA; 4 Department of Bioengineering, University of California, Berkeley, CA 94720 USA; 5 Bioscience Division, Los Alamos National Laboratory, Los Alamos, NM 87545 USA; 6 Joint Institute for Biological Sciences, Oak Ridge National Laboratory, Oak Ridge, TN 37831 USA; 7 National Virtual Biotechnology Laboratory (NVBL), US Department of Energy, Washington, DC USA

**Keywords:** Structural biology, X-ray crystallography, Drug discovery

## Abstract

Severe acute respiratory syndrome-coronavirus 2 (SARS-CoV-2), which causes coronavirus disease 2019 (COVID-19), threatens global public health. The world needs rapid development of new antivirals and vaccines to control the current pandemic and to control the spread of the variants. Among the proteins synthesized by the SARS-CoV-2 genome, main protease (M^pro^ also known as 3CL^pro^) is a primary drug target, due to its essential role in maturation of the viral polyproteins. In this study, we provide crystallographic evidence, along with some binding assay data, that three clinically approved anti hepatitis C virus drugs and two other drug-like compounds covalently bind to the M^pro^ Cys145 catalytic residue in the active site. Also, molecular docking studies can provide additional insight for the design of new antiviral inhibitors for SARS-CoV-2 using these drugs as lead compounds. One might consider derivatives of these lead compounds with higher affinity to the M^pro^ as potential COVID-19 therapeutics for further testing and possibly clinical trials.

## Introduction

In late December 2019, COVID-19 was reported in Hubei province, Wuhan, China, and it soon became a global pandemic affecting public health, social interactions, and economies^1,2^. The causative agent of COVID-19, SARS-CoV-2 is related to a group of positive-sense single-stranded RNA-containing coronaviruses which are pathogenic in vertebrates including humans^3^. Even though effective vaccines are now available, the need for the development of SARS-CoV-2-specific antivirals remains. The process of designing and developing new antiviral compounds can be lengthy. As an alternative, one approach is to search for existing approved drugs to repurpose. These repurposed drugs can then be minimally altered to increase their specificity to make effective SARS-CoV-2 antiviral therapeutics, thus expediting their approval for this new purpose.

The 30 kb single stranded RNA of the SARS-CoV-2 encodes four structural, sixteen non-structural (NS), and nine accessory proteins, 29 total, from fourteen open-reading frames (ORFs). Two of these ORFs, encode polyproteins (ORF1a and ORF1ab), that are proteolytically cleaved into sixteen non-structural proteins (NSP1-NSP16). The protein NSP3 is the papain-like protease (PL^pro^), and NSP5 is the main protease (M^pro^); they are responsible for processing the polyproteins. NSP3 and NSP5 are part of a larger replicase-transcriptase complex which includes other NSPs such as NSP7 and NSP8 (primase complex), NSP12 (RNA-dependent RNA polymerase—RdRp), NSP13 (helicase-triphosphatase), NSP14 (exoribonuclease), NSP15 (endonuclease), and NSP10 and NSP16 (methyltransferases). NSP2, NSP9, and NSP11 have unknown functions. Proteins NSP4 and NSP6 make a complex with NSP3^4^.

The structural proteins of the SARS-CoV-2 include spike (S), envelope (E), membrane (M), and nucleocapsid (N), which are involved in cell binding and fusion^5^, host recognition and virulence^6^, inhibiting interferon production^7^, and genome packaging^8^, respectively. The 3′ end of the genome expresses these four structural proteins as well as nine accessory proteins. The functions of most of the accessory proteins are unknown except for three of them, which are involved in inflammasome (Orf3a), as type I IFN antagonist (Orf6), and as suppressor of host antiviral response (Orf9b)^4^.

Almost all the SARS-CoV-2 proteins and their associated partner proteins, such as the type II transmembrane serine protease TMPRSS2, the adventitious partner of spike protein^9^, are potential targets for development of small molecule or biologic inhibitors. The first FDA-approved small molecule inhibitor for a SARS-CoV-2 protein was remdesivir, which was repurposed as an inhibitor of NSP12 (RdRp)^10^. Remdesivir was originally designed for combating Hepatitis C and subsequently the Ebola virus in 2014. Since NSP3 (PL^pro^) and NSP5 (M^pro^) are part of a larger RNA polymerase, some suggest that small molecule inhibitors of NSP3 and NSP5 will have synergistic effects to inhibitors of RNA polymerase such as remdesivir^11^.

The importance and efficacy of the non-structural NS3/4A protease inhibitors of Hepatitis C Virus (HCV) against SARS-CoV-2 has drawn much attention in the scientific community; many agree that they are effective inhibitors of SARS-CoV-2^12,13^. The α-ketoamide-containing covalent inhibitors are among lead candidates for binding to cysteine proteases, especially M^pro^^14–16^, because the two adjacent C=O groups create an especially powerful electrophile. Boceprevir was among the first Hepatitis C Virus antiviral agents to show inhibitory activity against M^pro^ and coronavirus^17–19^. Based on structural similarities of the HCV NS3/4A protease and M^pro^, several authors suggest that a variety of NS3/4A inhibitors would be effective against SARS-CoV-2 main protease^12,13^. Also, a structural analysis of binding of α-ketoamide inhibitors from experiments at room temperature suggests that a high degree of flexibility of the M^pro^ active site facilitates the binding of these antivirals^20,21^.

In this study, we attempted to obtain direct X-ray crystallographic binding evidence on six HCV NS3/4A protease inhibitors against SARS-CoV-2 main protease. Based on an initial molecular docking study on boceprevir, and a broad knowledge-based literature search, we selected boceprevir, telaprevir, narlaprevir, asunaprevir, grazoprevir, and simeprevir (Table S1) for co-crystallization trials. Also, we selected some other covalent binders (Table S2), mostly related to the family of small molecule cysteine protease and cathepsin inhibitors, for co-crystallization against M^pro^. Out of the other compounds incubated with M^pro^, only VBY-825 and leupeptin produced crystals suitable for X-ray crystallography.

## Materials and methods

### Cloning, expression, and purification of M^pro^

The codon-optimized synthetic gene of full length M^pro^ from SARS-CoV-2 was cloned into the pET29b vector carrying a C-terminal 6 × His-Tag sequence. The plasmid encoding M^pro^ was transformed into competent *E. Coli* BL21 (DE3) cells. Multiple colonies from the transformed plate were picked and incubated in the LB media containing 100 μg/ml kanamycin overnight at 37 °C. The cells were inoculated into 500 ml of auto induction ZYM 5052 medium and grown at 37 °C to an OD_600_ of about 0.6. The cells were then allowed to auto-induce^22^ overnight at 20 °C. The cells were harvested by centrifugation at 5000 rpm for 20 min, lysed at ice-cold temperature using bacterial protein extraction agent (B-PER, Thermo Fisher Scientific) in the presence of lysozyme and benzonase. The soluble and insoluble fractions were separated by centrifugation at 16,000 rpm for 25 min. The resulting cell-free supernatant was allowed to bind for 20 min at 20 °C with Ni–NTA agarose (thermo scientific) resin that had earlier been equilibrated with buffer A (40 mM Tris, 400 mM NaCl, 10 mM imidazole, pH 8.0). This mixture was then poured into a column and the resin was washed with 50 ml binding buffer. The protein was eluted using a step gradient with increasing concentration of imidazole (50, 100 and 250 mM). Fractions of the eluate were analyzed on 4–10% SDS–PAGE gel. Further purification was achieved by a size-exclusion (Superdex increase-200) column that had previously been equilibrated with a buffer containing 40 mM Hepes pH 7.4, 2 mM TCEP, and 300 mM NaCl. The histidine tag was cleaved by human rhinovirus (HRV) 3C protease (AcroBIOSYSTEMS) and further purified by reverse nickel-affinity chromatography. The purified protein was then dialyzed overnight at 4 °C against 30 mM Hepes pH 7.4, 200 mM NaCl, 1 mM TCEP, and concentrated to ~ 7 mg/ml and used for crystallization or stored at −80 °C.

### Compounds

Boceprevir, Telaprevir, Narlaprevir, Grazoprevir, Asunaprevir, Simeprevir, Leupeptin, VBY-825, Balicatib, CA-074, Calpeptin, E-64, E-64c, JPM-OEt, LY-3000328, Odanacatib, CPI (Cysteine Protease Inhibitor), Aloxistatin, Cinanserin, MDL-28170, MG-101, ONO-5334, (±)Alliin, and Z-LVG-CHN2 were purchased from MedChemExpress, LLC. (www.medchemexpress.com). DiscoveryProbe™ FDA-approved Drug Library (23 plates of 96-well, 1971 compounds) were purchased from APExBIO (https://www.apexbt.com).

### Protein crystallization

M^pro^ crystals were grown using either hanging drop or sitting drop vapor diffusion methods by manual and robotic methods. Crystallization conditions were 22% PEG 4000, 0.1 M Hepes pH 7.0, and 3% dimethyl sulfoxide (DMSO). Boceprevir, telaprevir and narlaprevir, were dissolved in 100% molecular biology grade DMSO and they were diluted to 1 mM solution using the crystallization well solution prior to the final drop setup with a 2:1, 1:1, and 1:2 ratio of M^pro^ (5.7–6.7 mg/ml) and the well solution containing the inhibitors at 1 mM concentration.

The first contact between the M^pro^ and HCV inhibitors was the crystallization drop. M^pro^-inhibitor complex plate-shaped crystals appeared three to five days after the crystallization setup. Leupeptin-containing (0.6 mM in final drop) M^pro^ crystals were grown using a 1:0.75 ratio of precipitant:M^pro^. For the VBY-825 M^pro^ complex, a final drop concentration of 1 mM VBY-825 and 4% DMSO was used in a 2:1 ratio of precipitant:M^pro^ due to the challenges posed by insolubility issues of VBY-825 in crystallization conditions.

Also, we used a High Throughput Acoustic Droplet Ejection (HT-ADE) method to grow M^pro^-telaprevir and M^pro^-boceprevir crystals. Crystallization drops were set-up with 40 nL of M^pro^ solution and 40 nL of the well solution containing 1 mM telaprevir or boceprevir followed by 2.5 nL of seed stock generated from apo M^pro^ crystals grown with conventional sitting drop vapor diffusion. HT-ADE co-crystallization attempts were also made for grazoprevir (1 mM), asunaprevir (1 mM), and simeprevir (0.5 mM), however no ligand was observed in electron density maps arising from these attempts.

For completeness we considered the following additional compounds as inhibitors for M^pro^ co-crystallization, which did not produce diffraction quality crystals: Balicatib, CA-074, Calpeptin, E-64, E-64c, JPM-OEt, LY-3000328, Odanacatib, CPI, Aloxistatin, Cinanserin, MDL-28170, MG-101, ONO-5334, (±)Alliin, and Z-LVG-CHN2.

For the FDA-approved drug library, we used an Opentrons high-precision OT-2 laboratory robot (https://opentrons.com/ot-2) to prepare the plates by dispensing 100 µL of 100% molecular biology grade DMSO to solubilize the ligands. We attempted the M^pro^ crystallization using 88 selected compounds (based on higher score hits calculated as binding affinity in kcal/mol from the molecular docking as well as their chemical reactivity towards cysteine) using the high throughput ADE method. In cases where sample identity could not be confirmed, or hits were duplicated (different salts of the same compounds), or the unavailability of the compounds, the hits were omitted from the crystallization trials. The final drop contained 40 nL M^pro^, 40 nL precipitant, 2.5 nL ligand and 2.5 nL seeding materials. Most of the selected compounds in this category did not produce diffraction-quality crystals or the ligand did not bind to the M^pro^.

### Data collection, structure determination and refinement

All data were collected at the 17-ID-1 (AMX) and 17-ID-2 (FMX) beamlines at the NSLS-II, Brookhaven National Laboratory (BNL), Upton, NY, United States. The energy of the X-ray beam was 12.66 keV (0.979 Å) at 17-ID-2 and 13.5 keV (0.920 Å) at 17-ID-1 beamline. To collect data at 100 K, we used Oxford cryosystems 800, and to capture the diffraction images we used Eiger 16 M and Eiger 9 M pixel array detectors from Dectris. Diffraction images were indexed, integrated, and scaled using *XDS*-based *FastDP*^23,24^. The Matthews coefficient (*V*_M_) was calculated as 2.02 Å^3^ Da^−1^, which corresponds to one monomer per asymmetric unit with an estimated solvent content of 39% (*e.g.*, PDB entry: 7K40). A summary of the data-collection statistics is shown in Tables S3 and S4.

We used *Phaser*^25^ (2.8.3) and *Dimple*^26^ (2.5.7) for molecular replacement and ligand search. We used *Refmac*^27^ (5.8.0) as implemented in *CCP4*^28^ (7.1.0) and *Phenix*^29–31^ (1.19.2) for refinement, and *Coot*^32^ (0.9.4) for model building. Refinement statistics and model validation values are shown in Tables S3 and S4. All molecular-graphics figures were created using *PyMOL* (v.2.4.1; by Schrödinger) (https://pymol.org).

### Molecular docking studies

We used *AutoDock Vina* (version 1.0) for docking studies (https://vina.scripps.edu)^33^ on three HCV NS3/4A protease inhibitors against SARS-CoV-2 M^pro^. We defined the minimum arguments required to run the docking program including the receptor, ligand, and search space arguments. We used a random seeding for the ligand which start the docking with a random conformation of the ligand. Exhaustiveness of the global search and number of binding modes were defined as 8 and 20, respectively. We used the following M^pro^ PDB entries with all the water and ligand molecules removed for the preparation of the receptors: 6WNP for boceprevir, 7C7P for telaprevir, and 6XQT for narlaprevir. We used *AutoDockTools* version 1.5.6 (http://mgltools.scripps.edu/) for preparing the ligand and receptor files and to define the search space. Receptor and ligand molecules were treated as rigid and as flexible, respectively.

Also, for virtual screening using high throughput molecular docking , we used a modified script (File S1 and File S2) to employ *AutoDock Vina* on small molecule databases. The original shell script is available on the *AutoDock Vina* website (https://vina.scripps.edu). We used ZINC15, a free database of commercially available compounds for virtual screening, for building our small molecule databases (https://zinc15.docking.org/)^34^. We compiled three databases: “fda” subset with 1426 compounds, “in-trials” subset with 6848 compounds, and “in-vitro” subset with 161,758 compounds. These databases were used for molecular docking against some of the SARS-CoV-2 targets. We used both personal computers and the National Synchrotron Light Source II High Performance Computing (NSLS-II HPC) AMX and FMX nodes for virtual screening of M^pro^ against the “fda” subset which contains a list of FDA-approved drugs. The name of the compounds, their crystallization status with the M^pro^ and some of the docking results are included in Tables S5 and S6, respectively.

### Microscale thermophoresis binding assays

Thermophoretic assays were carried out using a Microscale Thermophoresis Monolith NT.115 apparatus (NanoTemperTechnologies). We fluorescently labeled the target protein M^pro^ by coupling of M^pro^ lysine residues to *N*-hydroxysuccinimide of the dye NT647 (NanoTemper Technologies). We incubated M^pro^ and NT647 dye on ice in darkness for 30 min and separated fluorescently labeled M^pro^ from free dye by size-exclusion chromatography using a buffer composed of 30 mM sodium phosphate pH 8.0, 200 mM NaCl, 1 mM DTT. Labelling efficiency of M^pro^ was verified prior to performing the binding test and equilibrium dissociation constant (*K*_d_) determination. For the initial binding test, 200 nM fluorescently labeled M^pro^ was incubated with 50 μM of ligands (telaprevir and narlaprevir) for 15 min prior to detection. For *K*_d_ determination, 200 nM fluorescently labeled M^pro^ was incubated with serial dilution of telaprevir or narlaprevir for 15 min, before loading of approximately 5 μL of the samples into capillaries. Thermophoretic measurements were performed using 40% MST power and 80% LED power at 25 °C.

## Results

### M^pro^ and HCV protease inhibitor complexes

**Figure 1:**
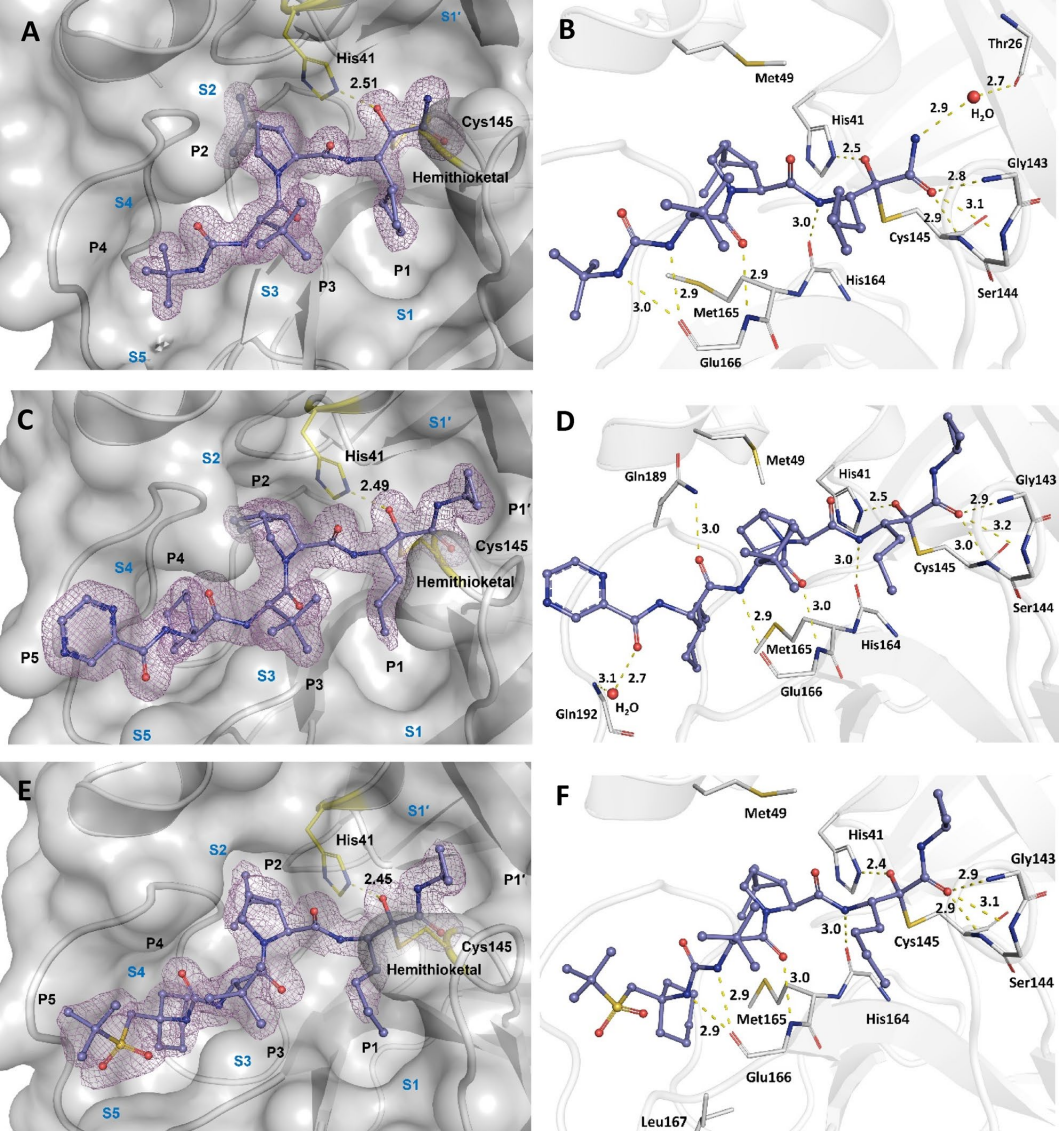
Active subsites of the M^pro^ binding pocket (S1′–S5), ligand functional groups or positions (P1′–P5), and hydrogen bonding interactions of the HCV NS3/4A inhibitors with M^pro^. (**A**) Electron density of the boceprevir complex at 1.35 Å resolution (PDB entry: 7K40). Boceprevir lacks the P1′ and P5 functional groups and therefore the associated enzyme subsites are not occupied. (**B**) Hydrogen bonding interactions of boceprevir complex. One structural water molecule facilitates the binding of the amine group of the α-ketoamide moiety. (**C**) electron density of the telaprevir complex at 1.48 Å resolution (PDB entry: 7K6D). (**D**) Hydrogen bonding interactions of the telaprevir complex. One structural water molecule facilitates the binding of the pyrazine (P5) moiety. (**E**) Electron density of the narlaprevir complex at 1.79 Å resolution (PDB entry: 7JYC). (**F**) Hydrogen bonding interactions of the narlaprevir complex. Ligands are shown as ball-and-stick representation. Electron densities (2F_o_–F_c_, 1 rmsd) are shown as purple mesh. All distances are in Å. (**C**) reproduced with permission of the International Union of Crystallography, Acta Cryst. (2021), A77, C194 (https://doi.org/10.1107/S0108767321094885).

Three Hepatitis C virus NS3/4A α-ketoamide protease inhibitors with a similar peptidomimetic scaffold (boceprevir, telaprevir, and narlaprevir) (Fig. S1A-C) can bind to the SARS-CoV-2 M^pro^ active site (Fig. 1). Electron densities show a high occupancy by these inhibitors, with very good shape complementarity to the M^pro^ active site. The six functional groups of the inhibitors (P1′, P1-P5) occupy the M^pro^ active subsites as they are available; boceprevir lacks the P5 functional group and its P1′ does not have a cyclopropyl substitution. The P1′ amide of all these inhibitors binds to the S1′ active subsite of the M^pro^ and their ketone group undergoes a nucleophilic attack by Cys145 of the enzyme to make a hemithioketal covalent linkage.

In all three inhibitors, the hemithioketal oxygen makes a strong and short distanced hydrogen bond (Low-Barrier H-Bond: LBHB^35^) to the His41 as follows: 2.51 Å in boceprevir, 2.49 Å in telaprevir, 2.45 Å in narlaprevir. Our observations are consistent with the longer hydrophobic substitutions at P1′ and P1 being able to render an electron-donation propensity which facilitates short hydrogen bond formation. However, it is in contrast with other detailed studies others reported^20^ (2.4 Å in boceprevir, 2.5 Å in telaprevir, 2.8 Å in narlaprevir). We speculate that the difference could be attributed to the data-collection temperature (100 K in this study vs. the previous study at 293 K) or different protonation states of the hemithioketal oxygen and His41 side chain.

The hydrogen bonding patterns (Fig. 1) of these peptidomimetic inhibitors with M^pro^ are similar, and depend in part on the presence of their specific functional groups (P1′, P1–P5). Some noticeable differences in the H-bonding patterns exist including involvement of a structural water for binding of the amide group of boceprevir (P1′) to the Thr26 main chain, the interaction of telaprevir’s P4 and P5 carbonyls with Gln189 and Gln192 side chain and main chain (via H_2_O), respectively, and no water-mediated interaction for narlaprevir binding. Telaprevir is the only inhibitor that interacts with Gln189.

### Microscale thermophoresis binding assays

**Figure 2:**
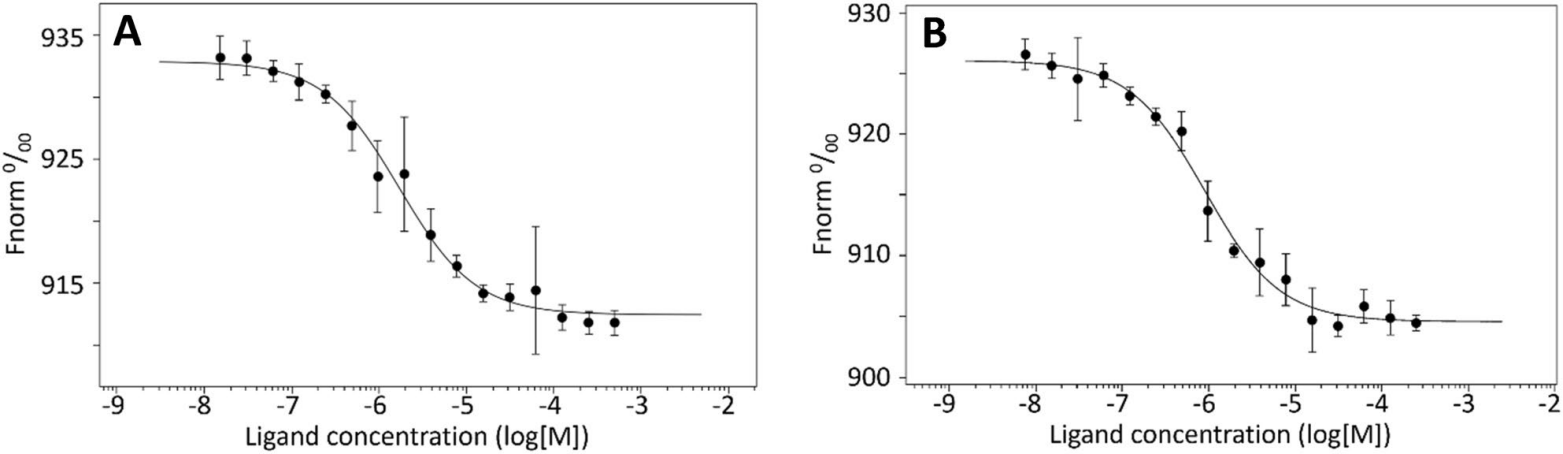
The equilibrium dissociation constants between (**A**) M^pro^-telaprevir (23 ± 4 μM) and (**B**) M^pro^-narlaprevir (12 ± 3 μM) were determined by thermophoretic experiments titrated against fluorescently labeled M^pro^. Dissociation curves were fit to the data to calculate the *K*_d_s. Data shown are the mean ± SD, n = 3. For boceprevir, the binding experiment was not successful due to the precipitation of the ligand in the assay buffer (30 mM sodium phosphate pH 8.0, 200 mM NaCl, 1 mM DTT). The precipitation led to turbidity and a non-uniform fluorescent signal.

The equilibrium dissociation constants (*K*_d_) between M^pro^-telaprevir (23 ± 4 μM) (Fig. 2A) and M^pro^-narlaprevir (12 ± 3 μM) (Fig. 2B) were determined by thermophoretic experiments in which the drugs were titrated against fluorescently labeled M^pro^. Our measured dissociation-constant values are consistent with the reported IC_50_, EC_50_, or binding constants within the margin of error *i.e.*, in the low µM binding range for these inhibitors (Table 1)^13,17,20,36–38^. For boceprevir, binding experiments were not feasible due to the poor solubility of the ligand.

**Table 1:** *K*_d_, IC_50_, and EC_50_ values for SARS-CoV-2 M^pro^-inhibitor complexes from inhouse and other studies.

Inhibitor	*K*_d_ (µM)	IC_50_ (µM)	EC_50_ (µM)
Boceprevir	^#^ND	0.95^38^ 3.1^20^ 2.7 ± 0.05^13^ 8.0 ± 1.5^17^	14.13^36^
Telaprevir	^#^23 ± 4	15.2^38^ 18^20^ 10.7 ± 0.4^13^	
Narlaprevir	^#^12 ± 3	1.1^38^ 5.1^20^ 16.11 ± 2.45^36^	7.23^36^
VBY-825	^#^ND		0.3^37^
Leupeptin	^#^ND	92^20^	

*ND* not determined.

^#^This study.

### M^pro^ and other inhibitor complexes

**Figure 3:**
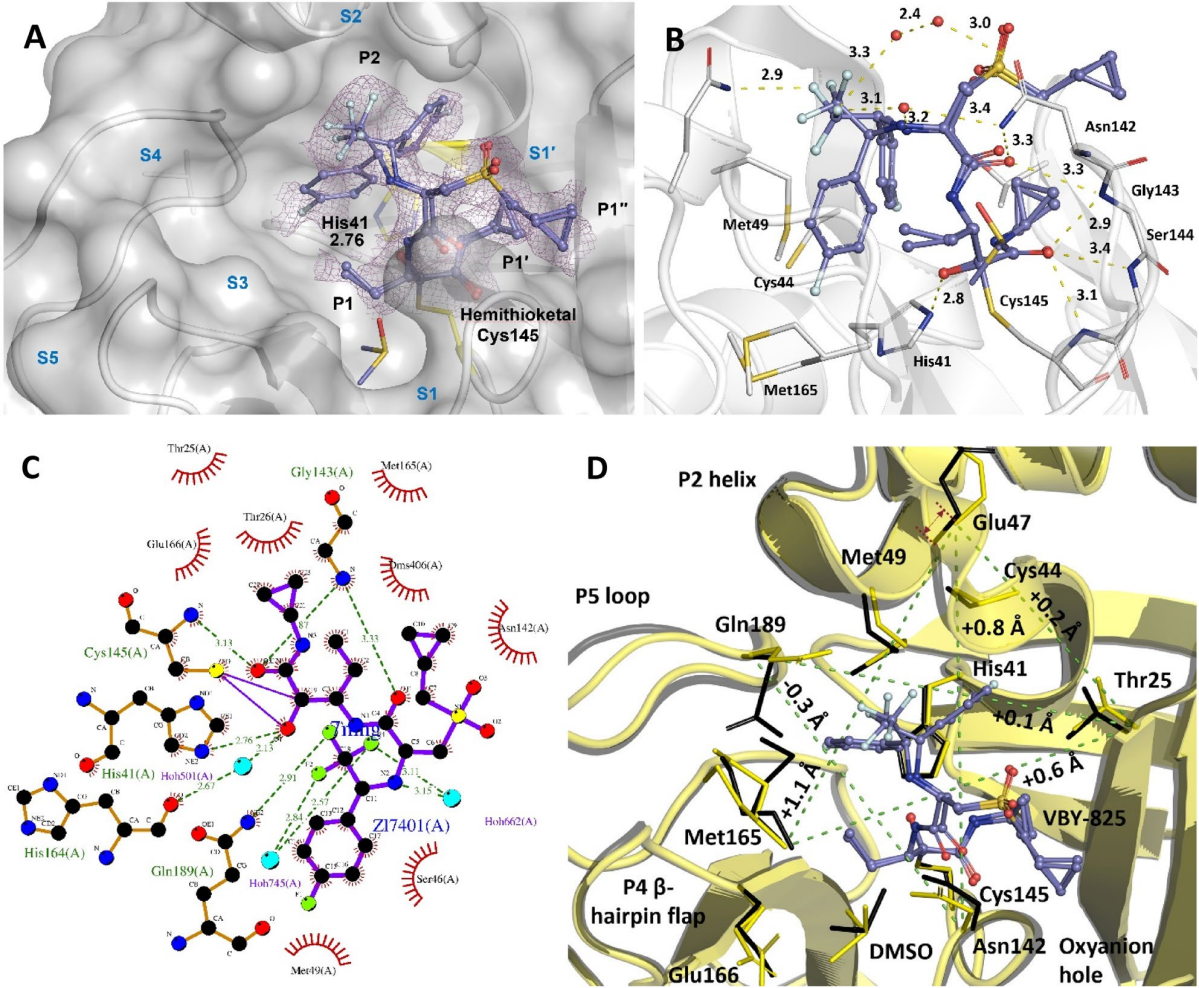
Active subsites of the M^pro^ binding pocket (S1′–S5), ligand functional groups (P1, P1′, P1′′, & P2), and hydrogen bonding interactions of the novel covalent inhibitor VBY-825 with M^pro^. Two different conformations of VBY-825 are shown. (**A**) Partial electron density of the VBY-825 M^pro^ complex at 1.70 Å resolution and 0.5 rmsd (PDB entry: 7MNG). VBY-825 lacks the P3–P5 functional groups and therefore the associated enzyme subsites are not occupied. Binding of a DMSO molecule to the S1 site forces the VBY-825 to bind in a tilted conformation to Cys145 (**B**) Hydrogen bonding interactions of VBY-825 M^pro^ complex shown for conformer A. Structural water molecules may facilitates the binding of the highly polarized fluorine groups of the trifluoromethyl moiety. A charge-assisted H-bond (CHAB)^35^ between H and F is possible, albeit not optimal. (**C**) A detailed 2D LigPlot+^53^ diagram of all the molecular interactions between conformer A of the VBY-825 and M^pro^ is shown. Hydrogen bonds are shown as green dotted lines. Spoked arcs represent nonbonded contacts such as hydrophobic interactions. The solid purple line between Cys145 and VBY-825 represents a covalent binding. (**D**) An overlay (0.28 Å r.m.s.d.) of the apo (black) (PDB: 7K3T) and VBY-825 bound (yellow) (PDB: 7MNG) M^pro^. The comparative changes in C_α_ distances are shown as green dotted lines with values. Binding of the VBY-825 displaces the P2 helix by 1.1 Å (red arrows) and predominantly displaces the side chains of Gln189 and Met165. However, the conformational changes are less significant in comparison with other HCV NS3/4A inhibitor-M^pro^ complexes^20^.

In the case of the VBY-825 molecule (Fig. 3A–C), which has the same α-ketoamide functional group as the other three HCV inhibitors, the binding pattern of the ketone is like that of the HCV inhibitors. However, the shape complementarity of the rest of the molecule, in terms of binding to the active site as well as the occupancy level, seemed sub-optimal. We speculate that the geometry of the molecule and its low solubility under the crystallization conditions may contribute to the partial occupancy at the active site.

In the complex with VBY-825, the S3-S5 binding sub-sites of M^pro^ are vacant and its cyclopropylmethanesulfonyl moiety (P1′′) binds close to the S1′ sub-site. The bulky trifluoroethyl 4-fluorophenyl seems to be very mobile and binds with a low occupancy to the S2 subsite in two different conformations. The solvent DMSO, required to solubilize the VBY-825 ligand is at a concentration of 4% in the crystallization drop. DMSO binds to the S1 sub-site, forcing the VBY-825 covalent bond to Cys145 into a slightly sub-optimal geometry, and the short ethyl group (P1) of VBY-825 cannot fully displace the DMSO molecule (Fig. 3A).

**Figure 4:**
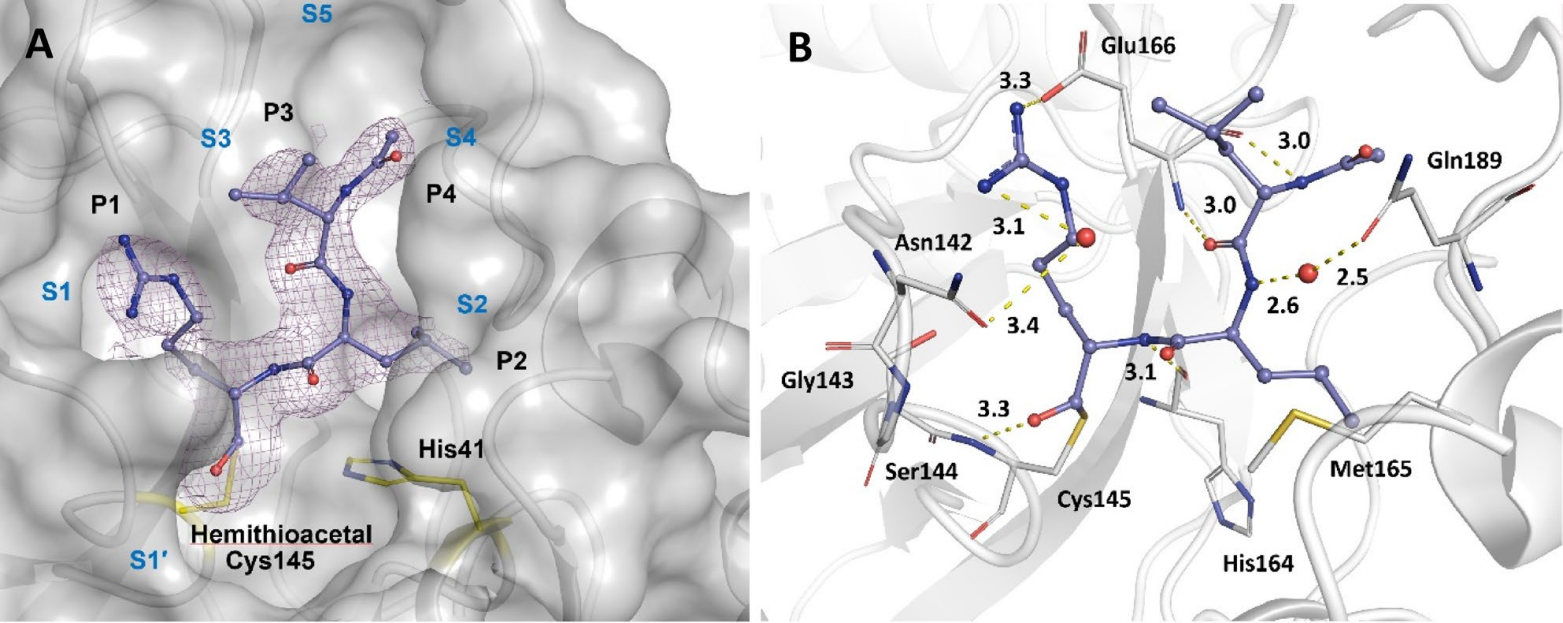
Active subsites of the M^pro^ binding pocket (S1′–S5), ligand functional groups (P1–P4), and hydrogen bonding interactions of the covalent inhibitor leupeptin with M^pro^. (**A**) electron density of the leupeptin M^pro^ complex at 2.3 Å resolution and 0.5 rmsd (PDB entry: 7MRR). Leupeptin lacks the P5 functional group and therefore the associated enzyme subsite is not occupied. (**B**) hydrogen bonding interactions of leupeptin M^pro^ complex shown. Two structural water molecules shown facilitate the binding of leupeptin.

As shown in Fig. 4, the aldehyde group of the microbial peptide leupeptin stereo-specifically reacts with Cys145 to form a hemithioacetal as an S enantiomer and is hydrogen bonded to the main chain nitrogen of the Cys145. This observation is consistent with room temperature studies reported^20^. New in this study is the finding that the arginine moiety of the leupeptin seems to make a weak hydrogen bond with the Glu166 side chain. Also, two structural waters facilitate the binding of leupeptin to the Gln189 and Asn142 side chains (Fig. 4B). Leupeptin makes three additional hydrogen bonds to the M^pro^ main chain.

### Molecular docking studies

Molecular docking is often used to study and predict the binding modes of a ligand to a receptor molecule and is widely used for drug discovery. Even though the binding mode observed by X-ray crystallography is often among the most favored modes of binding, the most favorable binding-energy mode may not necessarily be observed in X-ray crystallographic studies^12^.

Our molecular docking studies show that *AutoDock Vina* could predict binding modes of the HCV NS3/4A inhibitors to M^pro^ that accurately reflect published X-ray crystal structures by others and in this study (Fig. S2). Establishing the accuracy of the modeled binding modes and the calculation of the binding affinities are necessary for comparing them with the newly designed derivative such as L551. The affinity of the binding was calculated as − 7.7, − 6.7, and − 8.3 kcal/mol for boceprevir, telaprevir, and narlaprevir, respectively. Since *AutoDock Vina* does not simulate the covalent binding of these inhibitors to their target, there is a slight coordinate shift between the X-ray structures and the docking binding modes (poses). The shift is more prominent near the Cys145 where covalent binding to α-ketoamide occurs. Also, the calculations of the binding affinity in these cases do not consider the affinity of covalent binding.

## Discussion

We have investigated the interaction between drugs already approved for treatment of hepatitis C with the main protease of SARS-CoV-2, and observe their binding in X-ray crystal structures. The existence of these drugs and others suggests a short path to creation of effective curative new antivirals that can help quell the current pandemic.

### M^pro^-inhibitor complexes

A major component in all the HCV NS3/NS4 inhibitor drugs (Table S1) is a pyrrolidine/proline-like ring moiety (a cyclopentane in case of simeprevir) which acts as a molecular scaffold for three to four other substitutions. X-ray crystallographic structures of the known complexes of these antiviral agents with M^pro^ (Table S1) show that the stereochemistry of covalent binding to the active site Cys145 usually places this ring in the S2 binding pocket of the M^pro^ near the hydrophobic residues of Met165 and Met49. Examination of the S2 binding pocket reveals that it can only accommodate a maximum combined substitution of three to four carbon or equivalent atoms at both positions 3 and 4 of the pyrrolidine ring. Boceprevir, telaprevir and narlaprevir are the only compounds that have small enough ring substitutions to fit into the S2 binding pocket of M^pro^.

During our studies, we attempted to co-crystallize M^pro^ with grazoprevir, asunaprevir, and simeprevir as selected inhibitors without any success. The reason these compounds were not found to bind to the active site was apparently the presence of a large substitution at position 3 or 4 of the pyrrolidine ring. With similar reasoning we anticipated that no M^pro^ complex will be observed using any other HCV NS3/NS4 inhibitors that have similar large substitutions in position 3 or 4 (see Table S1); therefore, we omitted this class of compounds from our crystallographic studies. Molecular docking studies on some of these untested compounds may seem to indicate a binding to the main protease active site^11^. Based on our observations, the *in-silico* predictions do not necessarily translate into observable binding in X-ray crystallographic studies. However, possibly removal of the large substitutions at positions 3 or 4 of the pyrrolidine ring on these untested compounds may show binding. The same reasoning might be true for variants of these untested compounds with a different stereochemistry which avoids positioning the larger substitution into the S2 binding pocket.

VBY-825 is a powerful cathepsin-specific (B, L, S, and V cathepsins) cysteine protease inhibitor^39^ with potent anti-tumor activity. Binding of VBY-825 to M^pro^ is particularly interesting due to its ability to inhibit cathepsins, making it a dual action inhibitor since host cell cathepsins are involved in SARS-CoV cell entry^40,41^. There is no information available on whether VBY-825 can inhibit furin^42^ and TMPRSS2 which are other host cell proteases facilitating SARS-CoV-2 cell entry^41^. VBY-825 binding to M^pro^ is mainly due to the highly electrophilic α-ketoamide functional group, resembling the other HCV NS3/4A inhibitors, and to the hydrophobic interactions (fluorophenyl moiety). Also, binding of VBY-825 to M^pro^ induces some conformational changes around the active site even though it does not occupy the S3-S5 subsites. However, the induced conformational changes at S3-S5 seem to be minimal (*e.g.,* P4 β-hairpin flap^20^). The P5 loop seems to be relatively mobile and the bulky fluorinated functional groups of VBY-825 exert a movement of 1.1 Å on the P2 helix (Fig. 3D). The r.m.s.d. between the apo (PDB: 7K3T) and VBY-825 bound (PDB: 7MNG) M^pro^ structures is 0.28 Å.

Leupeptin^43^ (a natural microbial peptide and protease inhibitor) forms a S-hemithioacetal with Cys145 through its aldehyde group. The PDB entry 7NEV^44^ for the leupeptin-M^pro^ complex shows two binding modes (R and S) in terms of the stereochemistry while 7MRR (this study) shows the S-enantiomer binding mode consistent with the reported room temperature studies (PDB entry 6XCH)^20^. As described in detail in room temperature studies^20^, the binding of leupeptin resembles the metastable tetrahedral intermediate of a protease, which eventually leads to the production of acyl intermediates.

### Significance of the M^pro^ inhibitors to human health

Successful introduction of the effective SARS-CoV-2 vaccines and antibodies for treatment of COVID-19 patients, does not necessarily reduce the significance and the urgency of developing small-molecule inhibitor drugs, because the virus continually mutates^45^. The likelihood of the mutations is high for structural proteins such as spike protein. Certain mutations in the spike protein may reduce the effectiveness of the vaccines and antibodies as the mutations occur^46^.

Non-structural proteins on the other hand, are less prone to mutations. A mutation in the NSP5 or M^pro^ active site may subsequently require multiple concerted mutations in its substrates. The likelihood of multiple concerted M^pro^ mutations accumulating fast enough to produce a resistant virus is much lower than mutations of a structural proteins such as the spike protein. For this reason, the inhibitors of the M^pro^, will likely be a more stable and effective solution in the long run. The same reasoning is true for other NSPs such as NSP3 or PL^pro^. Also, small molecule inhibitors are a better choice than vaccines for infected individuals and may work synergistically with neutralizing antibodies and remdesivir.

### The rationale behind new drug design

A detailed analysis of the M^pro^ active subsites in terms of their hydrophilicity in relation to the functional groups of the inhibitors^14,15,20^ suggests that an improvement in binding is possible. The P1 functional group occupying the S1 subsite is the most noticeable group for a change. The P1 chemical group in all the three HCV inhibitors is a cyclic/linear hydrophobic chain which occupies a fully hydrophilic S1 subsite. Conceivably, replacing the current P1 groups with a functional group capable of making a hydrogen bond with both side chains of the His163 and Glu166 will make these inhibitors more specific for a more efficient binding to SARS-CoV-2 M^pro^. Numerous PDB structures (*e.g.*, 7JT7^47^, 7LYH, 7LYI, 7CB7^48^) suggest that a pyrrolidone functional group, among others, is one of the best fits in binding to the S1 subsite. This change may significantly improve the binding of these inhibitors without significantly changing their ADME^49^ (Absorption, Distribution, Metabolism, and Excretion) properties and safety margins as FDA-approved drugs.

This approach would shorten safety testing and evaluation times, helping to fulfil the urgent need for new antiviral drugs. We proposed that three compounds, with a design based on the HCV NS3/4A inhibitors (L551, L737, and L751) (Fig. S1D-F) may bind more tightly to the M^pro^. While we were evaluating the viability of chemical synthesis and subsequent testing of these three new compounds for x-ray crystallography and binding studies, an experimental new drug by Pfizer (PF-07321332) (Fig. S1G) entered clinical trial^50–52^ (PDB IDs: 7SI9, 7VH8, 7RFS, 7RFW). The design of PF-07321332 was based on PF-00835231 (structurally similar to GC-376^18,54^); however, the final new drug is ultimately a pyrrolidone derivative of a boceprevir-like compound with a nitrile as an active functional group instead of α-ketoamide, which corroborates our hypothesis that pyrrolidone derivatives of HCV NS3/4A inhibitors are potentially good lead compounds for the design of new M^pro^ inhibitors. Also, the recently published structures of the M^pro^ with BBH-1, BBH-2, and NBH-2 shows that this approach can succeed^55^.

The M^pro^ S2 subsite is hydrophobic, and all the three inhibitors seem to be able to use this site for efficient binding using their hydrophobic P2 groups. The S3 and S5 binding subsites are shallow, and they lie at the surface of the M^pro^. Telaprevir can efficiently use both subsites, especially S5, by making a hydrogen bond to Gln189. The S4 subsite is amphiphilic, and therefore an amphiphilic P4 functional group might be a better choice for improving the binding. Also, a detailed recent study^55^ shows the importance of the protonation state of the His41 and the keto-warhead, as well as the role of oxyanion hole (comprised of Gly143, Ser144, and Cys145 main chain), in determining the binding affinity of the various inhibitors.

### Comparison with other similar ligands

**Figure 5:**
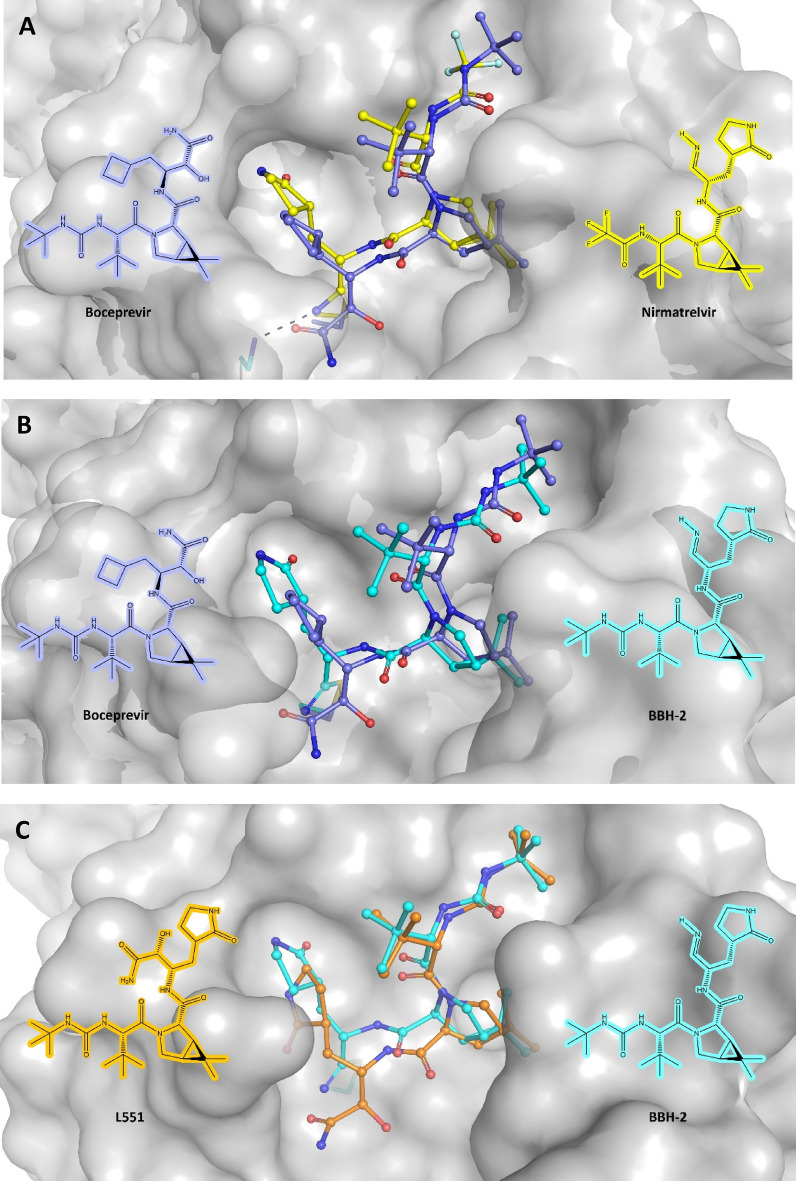
Comparison of the binding modes of four closely related ligands to the M^pro^. (**A**) Comparison of boceprevir (PDB: 7K40—this study) and nirmatrelvir^51^ (PDB: 7RFW) binding modes to the M^pro^. The nitrile warhead of the nirmatrelvir can make a 3.0 Å hydrogen bond to the Gly143 (shown in black dotted line) (**B**) comparison of boceprevir (PDB: 7K40) and BBH-2^55^ (PDB: 7TEH) binding modes to the M^pro^. (**C**) comparison of the designed L551 with the molecular docking binding mode as shown (Fig. S2D—this study) and the binding mode of BBH-2 (PDB: 7TEH) to the M^pro^. The chemical structures are shown in their binding form. Structures were drawn using ChemDraw 14 Professional from PerkinElmer (https://perkinelmerinformatics.com/products/research/chemdraw).

As shown in Fig. 5, we selected three ligands closely related to boceprevir (nirmatrelvir, BBH-2, and L551) to compare their structure and geometry of binding to the M^pro^. The main scaffold of these peptidomimetic inhibitors as well as the dimethyl-bicyclo[3.1.0] proline moiety is the same or very similar. The first major difference between boceprevir and Pfizer’s nirmatrelvir (PF-07321332)^51^ (Fig. 5A) and BBH-2^55^ (Fig. 5B) is the functional group (the warhead) that covalently binds to the Cys145 in the active site. The reactive functional groups of boceprevir and nirmatrelvir are ketoamide and nitrile, respectively. The second major difference is the presence of a γ-lactam 5-membered ring (pyrrolidone-like) as P1 chemical group which significantly increases the binding affinity by making additional hydrogen bonds^51,55^. The P1 group of boceprevir (a cyclobutyl moiety) cannot form any hydrogen bonds. Nirmatrelvir (PF-07321332) is the active ingredient of the orally bioavailable drug Paxlovid™ by Pfizer which is specifically approved for emergency use authorization (EUA) by the Food and Drug Administration (FDA) for the treatment of COVID-19 patients. The *K*_i_ and *K*_d_ of nirmatrelvir for main protease are 3 nM^51^ and 7 nM^55^, respectively, which are largely driven by the favorable enthalpy^55^ of the reaction with the main protease. The *K*_d_ value of the BBH-2/M^pro^ complex is 30 nM as reported^55^ which is comparable to (but less than) the values reported for nirmatrelvir.

Molecular docking suggests that the designed L551 inhibitor (this study) may bind to the main protease with the same geometry as BBH-2 (Fig. 5C). The only difference between L551 and BBH-2 is their reactive warhead group which are ketoamide and nitrile, respectively. As shown in Fig. 1A, boceprevir’s α-ketoamide warhead can make a total of at least three hydrogen bonds as shown: one with His41 (with correct protonation states), one with Thr26 via H_2_O, and the third one using its amide carbonyl which is within an easy hydrogen bond-making distance with all the N–H groups of the main chain of the oxyanion hole (Gly143-Ser144-Cys145 moieties). In case of the nitrile warhead (nirmatrelvir and BBH-2), it can make only one hydrogen bond to the oxyanion hole^55^. Since L551 has the same reactive functional group as boceprevir (ketoamide), we anticipate that the binding affinity of the L551 to the M^pro^ could be higher than, or at least within the same range as, that of the BBH-2/M^pro^ complex. Also, we anticipate that the affinity of a CF3-substituted derivative of L551 (L546) (Fig. S1J) maybe within the same range as the affinity of the nirmatrelvir to the M^pro^, if not better.

The importance of one additional hydrogen bond in increasing the affinity of a ligand to the M^pro^ is reported for the compound PF-00835231 (Fig. S1K) from Pfizer^51^, which has an inhibition constant (*K*_i_) of 0.27 nM for the main protease. The reactive warhead group of this compound can make at least one hydrogen bond with the oxyanion hole and one additional hydrogen bond with His41. The same compound with the nitrile warhead (Fig. S1L) can make at least one hydrogen bond only to the oxyanion hole (like nirmatrelvir – Fig. 5A) which reduces its *K*_i_ for the M^pro^ by 100-fold to 28 nM^51^.

## Conclusion

With the SARS-CoV-2 continuously mutating, the need for the design of novel M^pro^ inhibitors to battle the current and future COVID-19 outbreaks is more than ever. The original HCV protease inhibitors (boceprevir, telaprevir, and narlaprevir) are drugs already approved by FDA and others, and their pharmacokinetics (Table S7) suggest that an inhibitory human blood plasma concentration (C_max_) against M^pro^ can be reached especially for boceprevir and narlaprevir. Other studies have suggested that these original HCV inhibitors could be used in drug repurposing strategies in combination with other inhibitors^38^. However, their lower affinity to the M^pro^ may not justify their use in clinical trials against COVID-19 in terms of economic viability of the treatment options. Therefore, one might consider derivatives^51,55^ of these HCV inhibitors with a higher affinity to the M^pro^ for clinical trials as potential COVID-19 therapeutics. Together with broad vaccination campaigns, such treatment options may help significantly to curtail the morbidity and mortality of the current pandemic.

## Supplementary Information

Supplementary Information.

## Data Availability

All the atomic coordinates and their associated structure factors have been deposited to the Protein Data Bank (PDB) under the accession/entry codes of 7K6D, 7K6E, 7K40, 7K3T, 7JYC, 7MNG, and 7MRR.
