# Supplementary material for: Hepatitis C virus NS3/4A inhibitors and other drug-like compounds as covalent binders of SARS-CoV-2 main protease
Source: Sci Rep. 2022 Jul 16;12:12197. doi: 10.1038/s41598-022-15930-z (PMC9287821; doi:10.1038/s41598-022-15930-z)
Supplement: Supplementary file 1 — Supplementary Information. [file 41598_2022_15930_MOESM1_ESM.pdf]

Hepatitis C Virus NS3/4A Inhibitors and Other Drug-Like Compounds as Covalent Binders of SARS-CoV-2 Main Protease

Babak Andi[1,7*], Desigan Kumaran[2,7*], Dale F. Kreitler[1], Alexei S. Soares[1], Jantana Keereetaweep[2], Jean Jakoncic[1], Edwin O. Lazo[1], Wuxian Shi[1], Martin R. Fuchs[1], Robert M. Sweet[1], John Shanklin[2], Paul D. Adams[3,4,7], Jurgen G. Schmidt[5,7], Martha S. Head[6,7], Sean McSweeney[1,2,7*]

[1]Center for BioMolecular Structure, NSLS-II, Brookhaven National Laboratory, Upton, NY 11973, United States. [2]Biology Department, Brookhaven National Laboratory, Upton, NY 11973, United States. [3]Molecular Biophysics and Integrated Bioimaging Division, Lawrence Berkeley National Laboratory, Berkeley, CA 94720, United States. [4]Department of Bioengineering, University of California, Berkeley, CA 94720, United States. [5]Bioscience Division, Los Alamos National Laboratory, Los Alamos, NM 87545, United States. [6]Joint Institute of Biological Sciences, Oak Ridge National Laboratory, Oak Ridge, TN 37831, United States. [7]National Virtual Biotechnology Laboratory (NVBL), US Department of Energy, Washington, DC, United States.

[*]email: bandi@bnl.gov; kumaran@bnl.gov; smcsweeney@bnl.gov

Supplementary information

Figures

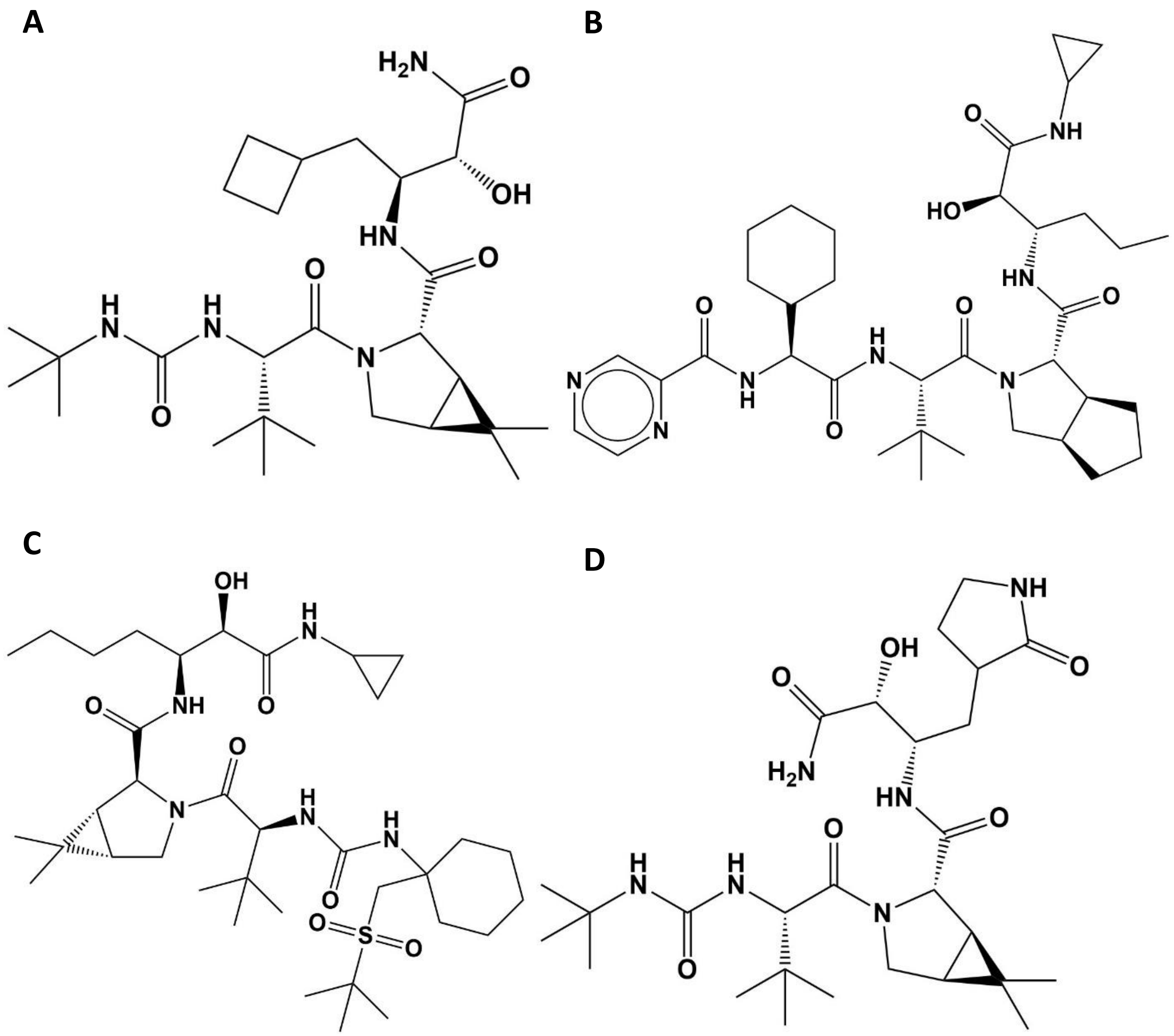

E

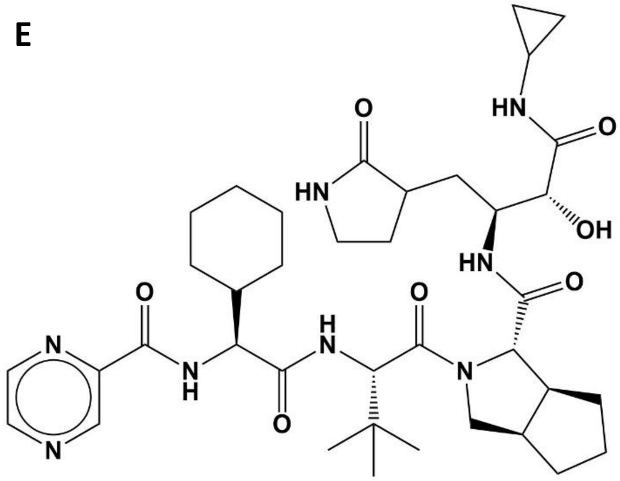

O
HN
HN
O
OH
HN
O
O
N
N
HN
H
O
O
H
N
N
O

F

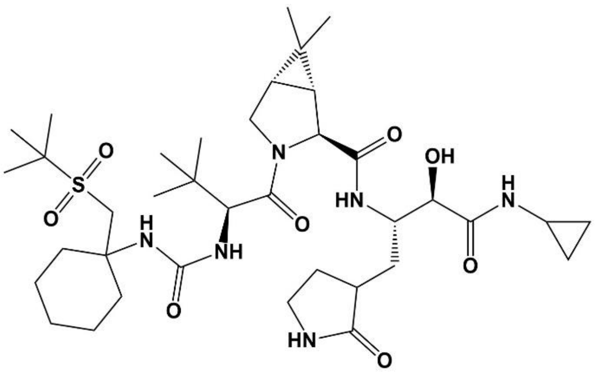

O
S
O
H
N
NH
O
N
O
HN
O
OH
H
N
O
HN
O

G

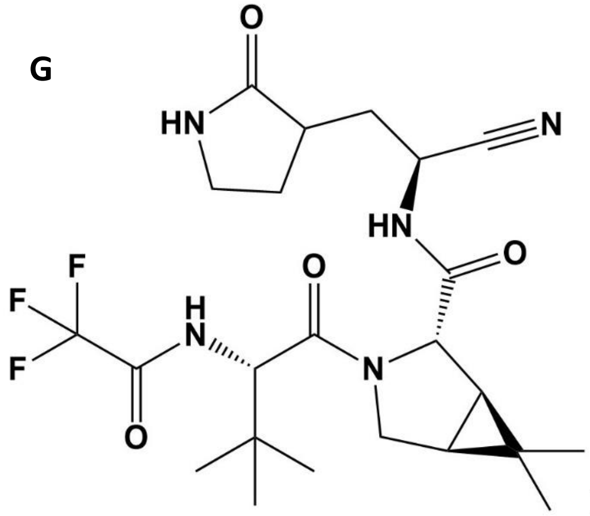

O
HN
N
HN
O
F
F
F
H
N
O
O
N

H

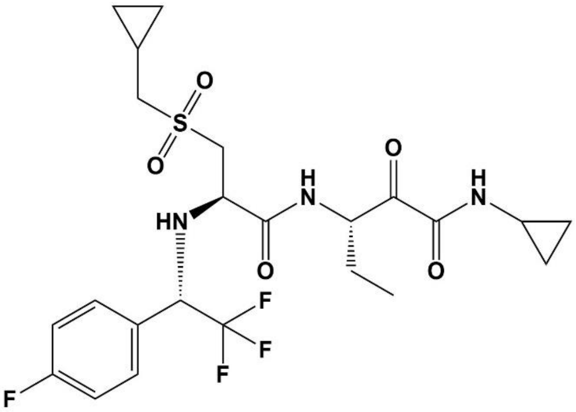

O
S
O
HN
H
N
O
O
O
H
N
F
F
F
F

I

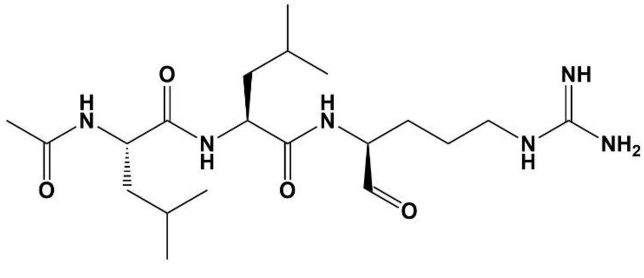

H
N
O
O
N
H
O
H
N
O
NH
H
N
NH2

J

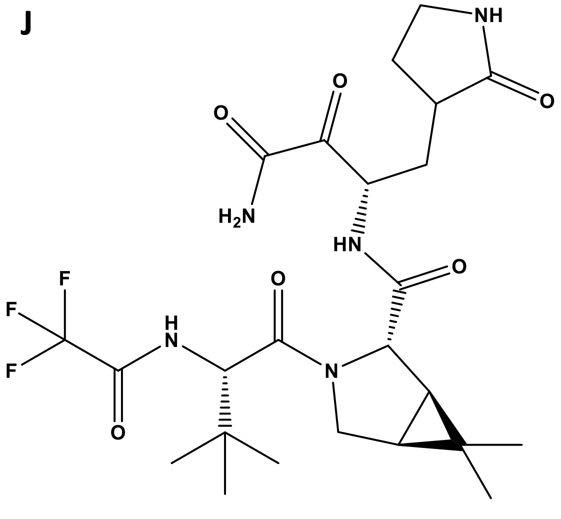

NH
O
O
O
H2N
HN
O
F
F
F
H
N
O
O
N

K

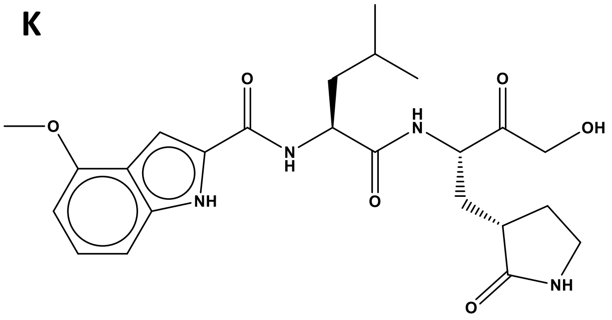

L

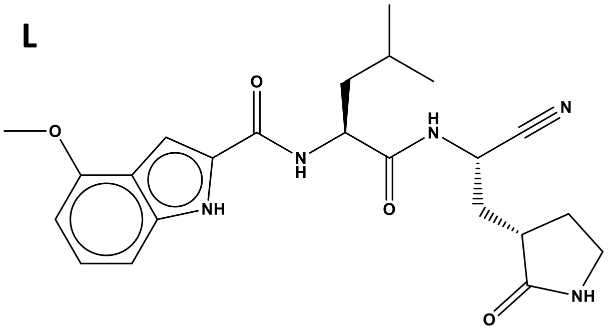

Fig. S1: Chemical structures of compounds with a potential to be orally administered as an inhibitor of SARS-CoV-2 M^{pro}. (A), chemical structure of boceprevir (bound form). (B), chemical structure of telaprevir (bound form). (C), chemical structure of narlaprevir (bound form). (D), 2-pyrrolidone derivative of boceprevir in bound form (L551). (E), 2-pyrrolidone derivative of telaprevir in bound form (L737). (F), 2-pyrrolidone derivative of narlaprevir in bound form (L751). (G), An experimental drug (PF-07321332) (a derivative of boceprevir and GC376) by Pfizer to enter clinical trials as the first orally administered compound for M^{pro} inhibition. (H), chemical structure of VBY-825. (I), chemical structure of leupeptin. (J), CF3-substituted derivative of L551 (L546). (K), chemical structure of PF-00835231 from Pfizer. (L), a derivative of the PF-00835231 with a nitrile reactive warhead. Structures were drawn using ChemDraw 14 Professional from PerkinElmer (https://shopinformatics.perkinelmer.com/search).

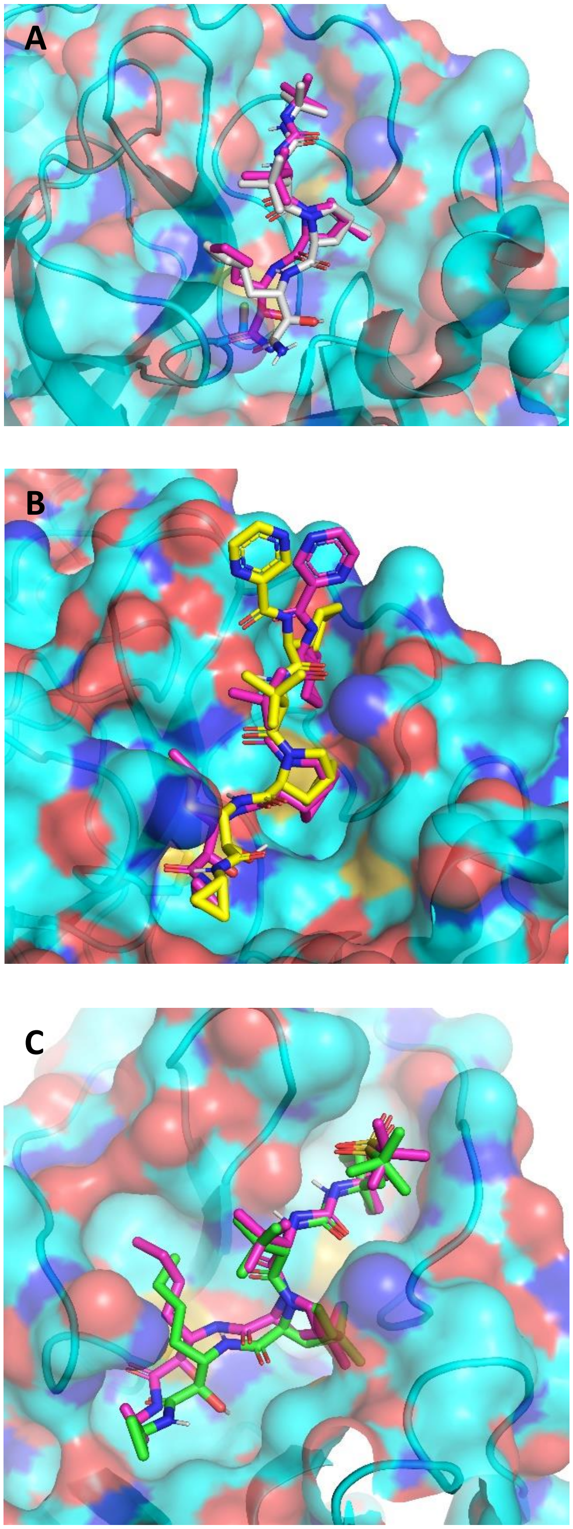
A
B
C

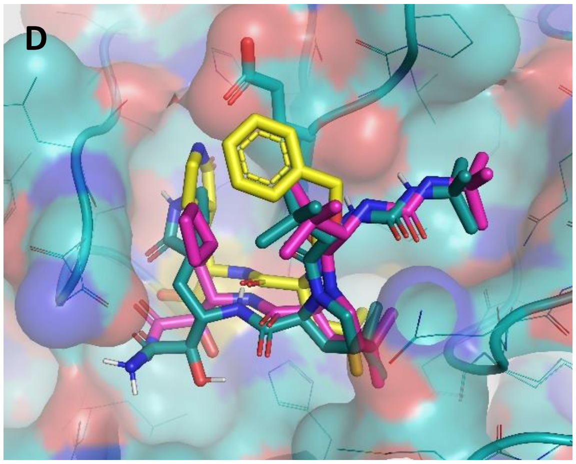

Fig. S2: Molecular docking of boceprevir, telaprevir and narlaprevir against SARS-CoV-2 M^{pro}. Protein molecules and the ligands were treated as rigid and flexible, respectively. (A), boceprevir molecule is shown as purple (experimental X-ray – PDB entry: 6WNP) and as gray (molecular docking with calculated affinity of -7.7 kcal/mol). (B), telaprevir molecule is shown as purple (experimental X-ray – PDB entry: 7C7P) and yellow (molecular docking with calculated affinity of -6.7 kcal/mol). (C), narlaprevir molecule is shown as purple (experimental X-ray – PDB entry: 6XQT) and green (molecular docking with calculated affinity of -8.3 kcal/mol). (D), boceprevir molecule is shown as purple (experimental X-ray – PDB entry: 6WNP), GC376 is shown in yellow (experimental X-ray – PDB entry: 7C6U), L551 (new design) molecular docking is shown in dark green with calculated affinity of -8.7 kcal/mol.

Tables

Table S1: HCV NS3/4A (–previr) family of protease inhibitors against SARS-CoV-2 M^{pro}.

Compound	M^{pro} binding observed (X-ray)	PDB entry codes against M^{pro}
Boceprevir	Yes[†]	7K40[#], 7C6S, 7COM, 6ZRU, 6XQU, 7BRP, 6WNP, 7NBR
Telaprevir	Yes[†]	7K6D[#], 7K6E[#], 6ZRT, 7C7P, 6XQS, 7NBS, 7LB7
Narlaprevir	Yes[†]	7JYC[#], 7D1O, 6XQT
Grazoprevir	No[#]	N/A
Asunaprevir	No[#]	N/A
Simeprevir	No[#]	N/A
Sovaprevir	NT	N/A
Ciluprevir	NT	N/A
Danoprevir	NT	N/A
Faldaprevir	NT	N/A
Glecaprevir	NT	N/A
Paritaprevir	NT	N/A
Vaniprevir	NT	N/A
Vedroprevir	NT	N/A
Voxilaprevir	NT	N/A

[†]Observed in this study and other studies.
[#]This study.
NT: Not Tested.
N/A: Not Applicable (No PDB entry found).

Table S2: Other small molecule cysteine protease and cathepsin inhibitors used in our studies.

Compound	MedChemExpress hyperlink
Leupeptin	www.medchemexpress.com/Leupeptin-hemisulfate.html
VBY-825	www.medchemexpress.com/VBY-825.html
Balicatib	www.medchemexpress.com/Balicatib.html
CA-074	www.medchemexpress.com/CA-074.html
Calpeptin	www.medchemexpress.com/Calpeptin.html
E-64	www.medchemexpress.com/E-64.html
E-64c	www.medchemexpress.com/Loxistatin_acid.html
JPM-OEt	www.medchemexpress.com/jpm-oet.html
LY-3000328	www.medchemexpress.com/LY-3000328.html
Odanacatib	www.medchemexpress.com/Odanacatib.html
Cysteine Protease Inhibitor	www.medchemexpress.com/Cysteine-Protease-inhibitor.html
Aloxistatin	www.medchemexpress.com/Aloxistatin.html
Cinanserin	www.medchemexpress.com/cinanserin-hydrochloride.html
MDL-28170	www.medchemexpress.com/MDL-28170.html
MG-101	www.medchemexpress.com/MG-101.html
ONO-5334	www.medchemexpress.com/ono-5334.html
(±)Alliin	www.medchemexpress.com/racemic-alliin.html
Z-LVG-CHN2	www.medchemexpress.com/z-lvg-chn2.html

Table S3: Data processing and refinement statistics for SARS-CoV-2 M^{pro} in complex with HCV NS3/4A covalent inhibitors.

PDB entry	7K40	7K6D	7JYC	7K6E
Ligand (inhibitor)	Boceprevir	Telaprevir	Narlaprevir	Telaprevir
Temperature (K)	100	100	100	100
Synchrotron	NSLS-II	NSLS-II	NSLS-II	NSLS-II
Beamline	17-ID-2	17-ID-1	17-ID-2	17-ID-1
Detector (Dectris)	Eiger 16M	Eiger 9M	Eiger 16M	Eiger 9M
**Data collection**				
Wavelength (Å)	0.979	0.920	0.979	0.920
Space group	*C*2	*C*2	*C*2	*C*2
a, *b*, *c* (Å)	114.3, 53.3, 45.8	109.7, 54.8, 48.2	113.2, 52.5, 45.7	109.6, 54.7, 48.0
α, β, γ (°)	90, 101.7, 90	90, 101.2, 90	90, 102.7, 90	90, 101.2, 90
Resolution (Å)	27.99-1.35 (1.38-1.35)	53.83-1.48 (1.51-1.48)	27.62-1.79 (1.83-1.79)	53.75-1.63 (1.63-1.66)
Unique reflections	59261 (3965)	46900 (2290)	24134 (1191)	34940 (1827)
R_{merge}	0.045 (1.243)	0.135 (3.00)	0.079 (1.199)	0.136 (3.347)
R_{pim}	0.02 (0.619)	0.045 (1.173)	0.047 (0.732)	0.044 (1.091)
$CC_{1/2}$	0.999 (0.505)	0.997 (0.352)	0.998 (0.545)	0.998 (0.368)
$I/\sigma I$	16.4 (1.00)	7.7 (1.2)	10.5 (1.3)	9.2 (0.7)
Completeness (%)	99.2 (89.9)	100.0 (100.0)	98.0 (79.8)	100.0 (99.9)
Redundancy	5.6 (4.7)	10.0 (7.2)	3.7 (3.3)	10.3 (10.3)
**Refinement**				
R_{work}/R_{free}	0.1698/0.1915	0.1847/0.2153	0.1750/0.2125	0.2141/0.2453
Favoured (%)	98.0	98.7	98.4	98.4
Allowed (%)	2.0	1.3	1.3	1.3
Outliers (%)	0.0	0.0	0.3	0.3
Bond length (Å)	0.015	0.008	0.010	0.007
Bond angles (°)	1.989	1.086	1.625	0.899

Table S4: Data processing and refinement statistics for SARS-CoV-2 M^{pro} in apo form and in complex with covalent inhibitors VBY-825 and leupeptin.

PDB entry	7MNG	7MRR	7K3T
Ligand (inhibitor)	VBY-825	Leupeptin	Zn^{2+} (Apo)
Temperature (K)	100	100	100
Synchrotron	NSLS-II	NSLS-II	NSLS-II
Beamline	17-ID-2	17-ID-2	17-ID-2
Detector (Dectris)	Eiger 16M	Eiger 16M	Eiger 16M
**Data collection**			
Wavelength (Å)	0.979	0.979	0.979
Space group	*C*2	*C*2	*I*2
a, *b*, *c* (Å)	114.7, 53.9, 44.8	113.2, 53.2, 45.9	44.8, 52.9, 111.3
α, β, γ (°)	90, 101.1, 90	90, 102.2, 90	90, 100.1, 90
Resolution (Å)	28.14-1.70 (1.70-1.73)	27.65-2.32 (2.32-2.38)	27.40-1.20 (1.22-1.20)
Unique reflections	29633 (1575)	11449 (667)	78254 (2561)
R_{merge}	0.184 (2.488)	0.115 (1.081)	0.094 (0.869)
R_{pim}	0.059 (0.834)	0.051 (0.481)	0.042 (0.428)
$CC_{1/2}$	0.997 (0.608)	0.997 (0.636)	0.997 (0.673)
$I/\sigma I$	9.0 (1.60)	12.1 (1.5)	11.6 (1.6)
Completeness (%)	100.0 (100.0)	98.3 (79.2)	97.8 (64.5)
Redundancy	10.3 (9.5)	6.4 (5.8)	6.2 (4.4)
**Refinement**			
R_{work}/R_{free}	0.1795/0.2179	0.1940/0.2455	0.1350/0.1670
Favoured (%)	98.7	96.1	95.3
Allowed (%)	1.3	3.9	4.7
Outliers (%)	0.0	0.0	0.0
Bond length (Å)	0.012	0.008	0.009
Bond angles (°)	1.701	1.537	1.694

Table S5: Compounds selected for crystallization trials on 96 well plate based on molecular docking of M^{pro} against the subset of FDA-approved drugs database. The numbers in bold are the product numbers as in DiscoveryProbe™ FDA-approved Drug Library (23 plates of 96-well) from APExBIO (https://www.apexbt.com). Boceprevir and Telaprevir compounds from the same library were included in crystallization trials as a control experiment (*a1* and *a2*). Conditions that produced crystals are highlighted in blue-gray background; however, only some of them had a sufficiently diffracting crystal for a definitive structure determination. Ligand names in green indicates that a bound ligand was observed. Ligand names in red indicates that ligand binding was inconclusive.

	1	2	3	4	5	6
a	**A3261** Boceprevir	**A4031** Telaprevir (VX-950)	**A2149** Bosutinib (SKI-606)	**A2168** Dovitinib (TKI-258, CHIR-258)	**A2451** Epirubicin HCl	**A2476** Idarubicin HCl
b	**A3145** Afatinib dimaleate	**A3222** Baricitinib phosphate	**A3381** Edoxaban	**A3397** Erlotinib	**A3513** Ixabepilone	**A3593** Mefloquine hydrochloride
c	**A4111** VX-680 (MK-0457,Tozasertib)	**A4135** Tofacitinib (CP-690550) Citrate	**A4138** Tofacitinib (CP-690550,Tasocitinib)	**A4154** Olaparib (AZD2281, Ku-0059436)	**A4327** Tadalafil	**A4379** Lonafarnib
d	**A8319** Dacomitinib (PF299804, PF299)	**A8322** Neratinib (HKI-272)	**A8325** Tivantinib (ARQ 197)	**A8334** Linsitinib	**A8351** Lumacaftor (VX-809)	**A8378** Betamethasone Dipropionate
e	**A8549** LY335979 (Zosuquidar 3HCL)	**A8650** Saxagliptin	**B1412** Flunarizine 2HCl	**A1670** Enzastaurin (LY317615)	**A2133** Saracatinib (AZD0530)	**B1477** Lurasidone HCl
f	**B1893** Azlocillin sodium salt	**B1895** Besifloxacin HCl	**B1924** Deflazacort	**B1926** Dexamethasone acetate	**B1931** Dimethyl Fumarate	**B1971** Methacycline HCl
g	**B3309** Cyproheptadine hydrochloride	**B3433** Flavoxate hydrochloride	**B3490** Eptifibatide	**B4745** (S)-10-Hydroxycamptothecin	**B5827** Poziotinib	**B5970** Sanguinarine chloride
h	**N2771** Cepharanthine	**P10019** Prulifloxacin (Pruvel)	**N2250** Vinorelbine	**N2471** Berbamine hydrochloride		

	7	8	9	10	11	12
a	**A2586** Nafamostat Mesylate(FUT-175)	**A2614** Bortezomib (PS-341)	**A2942** Masitinib (AB1010)	**A3001** PCI-32765 (Ibrutinib)	**A3012** Ruxolitinib (INCB018424)	**A3018** Trametinib (GSK1120212)
b	**A3660** Nilotinib monohydrochloride monohydrate	**A3781** Ruxolitinib phosphate	**A3791** Saquinavir mesylate	**A3863** Tedizolid	**A3892** Triptonide	**A4073** Raltegravir (MK-0518)
c	**A5133** Irinotecan	**A5467** Ponatinib (AP24534)	**A5618** Daclatasvir (BMS-790052)	**A5793** Quizartinib (AC220)	**A8171** Entinostat (MS-275,SNDX-275)	**A8207** AZD6244 (Selumetinib)
d	**A8402** Conivaptan HCl	**A8418** Dovitinib Dilactic acid	**A8423** Enoxacin (Penetrex)	**A8496** Paliperidone	**A8512** Rifaximin (Xifaxan)	**A8525** Sotrastaurin (AEB071)

e	**B1496**<br>Icotinib	**B1551**<br>Mizolastine	**B1612**<br>Pancuronium dibromide	**B1689**<br>Capreomycin Sulfate	**B1781**<br>Lomefloxacin HCl	**B1786**<br>Meclizine 2HCl
f	**B2144**<br>Fluocinonide	**B2159**<br>Eltrombopag	**B2171**<br>Imatinib (STI571)	**B2248**<br>Ketanserin	**B2295**<br>Pirarubicin	**B2303**<br>Apatinib
g	**B5996**<br>Motolimod (VTX-2337)	**C3812**<br>Cefonicid (sodium salt)	**C4274**<br>Quinestrol	**N1490**<br>Brucine	**N1753**<br>Scutellarin	**N2275**<br>Protopine
h						

Table S6: Top 10 hits from M^{pro} molecular docking results (using PDB entry 6M0K) against FDA-approved drugs subset of ZINC database. Ligands highlighted in blue were used in crystallization trials. The most stable binding mode of the ligands are shown at the active site of the enzyme.

Affinity = -9.6 kcal/mol<br>ZINC000169289767<br>Trypan Blue	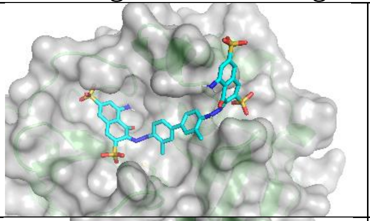	Affinity = -9.0 kcal/mol<br>ZINC000052955754<br>Ergotamine	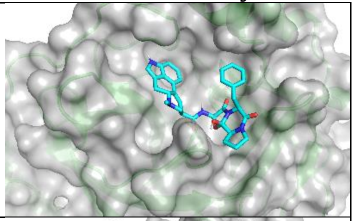
Affinity = -9.1 kcal/mol<br>ZINC000253632968<br>**Simeprevir**	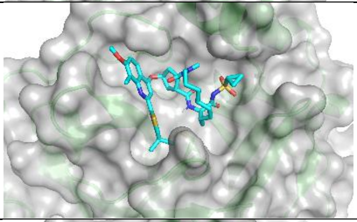	Affinity = -9.0 kcal/mol<br>ZINC000003978005<br>Dihydroergotamine	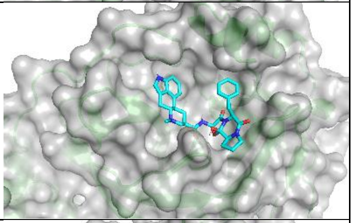
Affinity = -9.1 kcal/mol<br>ZINC000084668739<br>Lifitegrast	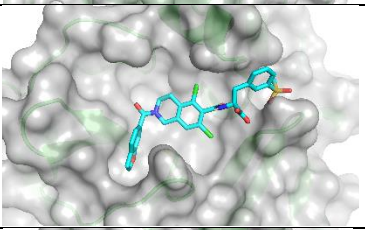	Affinity = -8.9 kcal/mol<br>ZINC000001612996<br>**Irinotecan**	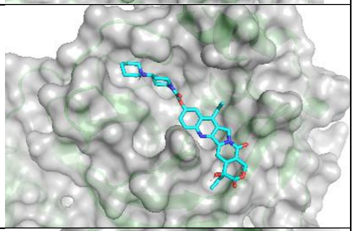
Affinity = -9.1 kcal/mol<br>ZINC000064033452<br>**Lumacaftor**	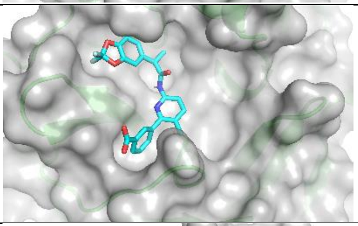	Affinity = -8.8 kcal/mol<br>ZINC000164760756<br>Olysio	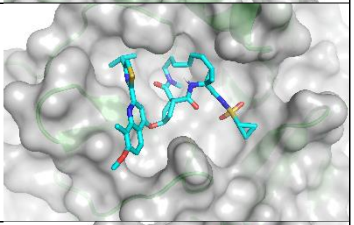
Affinity = -9.1 kcal/mol<br>ZINC000003932831<br>Avodart	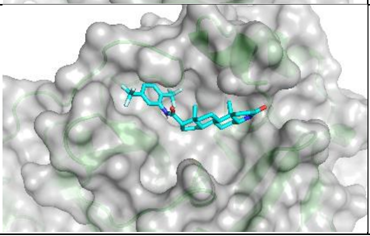	Affinity = -8.8 kcal/mol<br>ZINC000006716957<br>**Nilotinib**	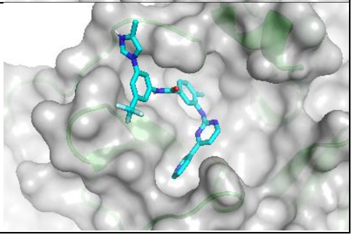

Table S7: Pharmacokinetics of HCV NS3/4A inhibitors. C_{max} (µM) values can be directly compared with the IC_{50} (µM) values (Table 1) for these inhibitors against SARS-CoV-2.

Inhibitor	Dose (mg)	C_{max} (µg/ml)	**C_{max} (µM)**	C_{min} (µg/ml)	AUC (µg·h/ml)	Reference
Boceprevir	800	1.72	**3.3**	0.09	5.41	FDA label for VICTRELIS
Telaprevir	750	3.51	**5.2**	2.03	22.3	FDA label for INCIVEK
Narlaprevir	200	0.36	**0.5**	N/A	1.92	doi:10.1128/AAC.01044-16
Narlaprevir + Ritonavir	100 100	1.2	***1.7**	N/A	14.3	

C_{max}: Peak plasma concentration after drug administration.
C_{min}: The lowest concentration before administering the next dose.
AUC: Area Under the Curve for integration of the concentration-time curve.
*Estimated as 6.8 µM for a 400 mg dose.

File S1: An example of a modified linux shell script to run *AutoDock Vina* on personal computers using a ZINC database for virtual screening. Users can modify the names shown in blue color.

```
#!/bin/bash

for f in ZINC*.pdbqt; do  b=`basename $f .pdbqt`; echo Processing ligand $b; mkdir -p XXXX;
./vina --config XXXX-1.txt --ligand $f --out XXXX/$f.pdbqt --log XXXX/$f.txt; done

#results

cd XXXX

grep "   1 " *.txt | cut -c1-16,35-45 >>result

sort -k 2 -r -o result.sorted result

cat result.sorted
```

File S2: An example of a modified linux shell script to run *AutoDock Vina* on HPC clusters using a ZINC database for virtual screening. Users can modify the names shown in blue color.

```
#!/bin/bash

num_procs=32

vinaRun () {
```

```
    local f=$1
    b=`basename $f .pdbqt`;
    echo Processing ligand $b;
    mkdir -p XXXX; /usr/local/crys-local/bin/vina --config XXXX-1.txt --ligand $f --out XXXX/$f.pdbqt --log XXXX/$f.txt
}

i=0
for f in ZINC*.pdbqt; do
    if (( i++ >= num_procs )); then
        wait -n
    fi
    vinaRun "$f" &
done

#results
cd XXXX
grep "   1 " *.txt | cut -c1-16,35-45 >>result
sort -k 2 -r -o result.sorted result
cat result.sorted
```
